# Asynchronous Recovery of Body Composition After Curative Surgery for Gastric and Colorectal Cancer: A Prospective Longitudinal Study †

**DOI:** 10.3390/nu18193172

**Published:** 2026-09-26

**Authors:** Jae Young Kang, Sang Hoon Kim, Da In Shin, Min Joon Kim, Woon Kyung Jeong, Seong Kyu Baek, Young Gil Son, Sung Uk Bae

**Affiliations:** 1 School of Medicine, Keimyung University, Daegu 42601, Republic of Korea; benjamink123@naver.com (J.Y.K.); dain000629@naver.com (D.I.S.); minjoon25@naver.com (M.J.K.); 2 Department of Surgery, School of Medicine, Keimyung University Dongsan Medical Center, Daegu 42601, Republic of Korea; ksh1944@gmail.com (S.H.K.); shinycloud@dsmc.or.kr (W.K.J.); sgbeak@dsmc.or.kr (S.K.B.); 3 Department of Surgery, School of Medicine, Inje University Paik Hospital, Busan 47392, Republic of Korea

**Keywords:** colorectal neoplasms, stomach neoplasms, body composition, bioelectrical impedance analysis, postoperative period

## Abstract

Background and Aims: Postoperative recovery of body composition in patients with gastric cancer (GC) and colorectal cancer (CRC) is a complex and dynamic process. However, longitudinal recovery patterns across multiple physiological domains remain incompletely characterized. This study aimed to describe longitudinal changes in body composition over 12 months after curative surgery in patients with GC and CRC. Methods: This prospective longitudinal study evaluated serial changes in body composition parameters, including body weight, skeletal muscle index (SMI), phase angle (PhA), and the extracellular water to total body water ratio (ECW/TBW), from the preoperative period to 12 months after curative surgery in 138 patients, comprising 61 patients with GC and 77 patients with CRC. Results: Distinct and time-dependent recovery patterns were observed across body composition domains. Body weight and SMI gradually declined after surgery, reaching their lowest levels at approximately 3 months postoperatively, followed by partial recovery at 6 and 12 months; however, complete restoration to preoperative values was not achieved in several subgroups. In contrast, PhA declined abruptly on postoperative day 1, while ECW/TBW peaked at the same time point, representing the most prominent early postoperative changes in these parameters. Descriptive subgroup patterns suggested less complete recovery of SMI, PhA, and ECW/TBW among elderly, low skeletal muscle mass, advanced-stage, and chemotherapy-treated patients. Patients with low skeletal muscle mass showed persistently lower SMI values, whereas advanced-stage and chemotherapy-treated patients appeared to show slower normalization of PhA and ECW/TBW. Conclusions: Postoperative changes in body composition appeared to occur asynchronously across multiple physiological domains in patients with gastric and colorectal cancer. Several clinically vulnerable subgroups, including older patients, patients with low skeletal muscle mass or advanced-stage disease, and patients who received chemotherapy, showed descriptive patterns suggestive of less complete recovery. Serial body composition assessment over an extended follow-up period may help characterize postoperative changes and provide a basis for future hypothesis-driven studies.

## 1. Introduction

Gastrointestinal malignancies, including gastric cancer (GC) and colorectal cancer (CRC), are among the most prevalent cancers worldwide and are frequently accompanied by profound metabolic and nutritional disturbances [1,2]. Beyond simple weight loss, cancer-related alterations in body composition—such as skeletal muscle depletion, impaired cellular integrity, and abnormal fluid distribution—have emerged as critical determinants of postoperative recovery, treatment tolerance, and overall clinical outcomes [3,4,5]. These multidimensional changes reflect the complex interplay between surgical stress, systemic inflammation, and cancer-related catabolism, underscoring the importance of comprehensive body composition assessment in patients undergoing curative surgery for gastrointestinal cancers.

Among available assessment tools, bioelectrical impedance analysis (BIA) has gained increasing attention as a practical, noninvasive, and cost-effective method for evaluating body composition in clinical settings [6,7]. BIA enables simultaneous assessment of multiple physiological domains, including skeletal muscle index (SMI), phase angle (PhA), and the extracellular water-to-total body water ratio (ECW/TBW). SMI reflects skeletal muscle mass relative to height, PhA is regarded as a surrogate marker of cellular integrity and membrane function, and ECW/TBW serves as an indicator of fluid balance and systemic stress [8,9]. These parameters are frequently altered in cancer patients, both as a consequence of the disease itself and following surgical intervention, including treatment with adjuvant chemotherapy [10]. They provide practical insights into nutritional status, cellular integrity, and fluid balance, making them valuable for monitoring changes in body composition from before surgery through postoperative recovery.

Despite growing recognition of the prognostic and clinical relevance of body composition, most previous studies have focused on short-term perioperative changes, typically comparing preoperative measurements with a single early postoperative time point [11,12,13,14]. While such approaches capture acute surgical stress, they fail to adequately reflect the dynamic recovery process that unfolds over several months, particularly during adjuvant treatment. Long-term trajectories of muscle mass, cellular health, and fluid balance remain insufficiently characterized, and existing longitudinal studies have largely examined gastric or colorectal cancer separately [10,15,16]. Consequently, comprehensive data describing postoperative body composition recovery across gastrointestinal malignancies are limited.

Therefore, the present study aimed to characterize longitudinal changes in body composition from the preoperative period to 12 months after surgery in patients with GC and CRC using serial BIA measurements. By repeatedly assessing body weight, SMI, PhA, and ECW/TBW at predefined time points, we sought to descriptively characterize postoperative changes over 12 months and explore numerical patterns across predefined clinical subgroups. A clearer understanding of these temporal patterns may provide a basis for future studies evaluating the potential clinical relevance of serial body composition assessment in patients with gastrointestinal cancer.

## 2. Materials and Methods

### 2.1. Study Design and Patients

This study was approved by the Institutional Review Board of Dongsan Medical Center (Daegu, Republic of Korea; IRB No. 2020-03-040), and written informed consent was obtained from all participants prior to inclusion. This was a prospective, longitudinal, observational study designed to evaluate postoperative changes in body composition in patients with gastrointestinal cancer. Patients with GC or CRC who underwent curative laparoscopic surgery at Dongsan Medical Center, Keimyung University, between July 2020 and July 2023 were consecutively screened for eligibility. Before enrollment, patients were excluded if they met any of the following predefined criteria: (1) presence of distant metastasis at diagnosis; (2) documented psychiatric disorders that could interfere with compliance with serial follow-up and BIA measurements; or (3) transfer to another hospital during the follow-up period. Eligible patients who met the inclusion criteria were prospectively enrolled and followed according to a predefined protocol that included serial BIA assessments. During the study period, a total of 144 patients were enrolled. Among these, patients who were subsequently lost to follow-up (*n* = 2), had insufficient data for analysis (*n* = 1), or refused continued participation during follow-up (*n* = 3) were excluded from the final analysis. After these post-enrollment exclusions, 138 patients were included in the final cohort. Of these, 77 patients had CRC and 61 had GC. The overall process of patient screening, enrollment, and exclusion is illustrated in Figure 1. Gastric and colorectal cancers were analyzed within a single cohort, as both represent major gastrointestinal malignancies associated with shared perioperative metabolic stress and nutritional challenges following curative surgical resection.

### 2.2. Data Collection and Definitions

All demographic, clinical, and treatment-related data were prospectively collected using a standardized case report form at the time of study enrollment and during scheduled follow-up visits. Collected variables included age, sex, body mass index (BMI), American Society of Anesthesiologists (ASA) physical status classification, preoperative C-reactive protein (CRP) levels, and the presence of preoperative intestinal obstruction. Pathological findings, including tumor stage and histologic characteristics, were determined based on postoperative pathology reports. Cancer staging was classified according to the American Joint Committee on Cancer (AJCC) staging system [17]. Patients were categorized by age (<65 years vs. ≥65 years) and cancer stage, which was dichotomized into early-stage disease (stage I–II) and advanced-stage disease (stage III–IV). Postoperative chemotherapy was recorded as a binary variable (received vs. not received). Nutritional status was assessed prospectively after cancer diagnosis and before surgery using the Patient-Generated Subjective Global Assessment (PG-SGA). The patient-generated component assessed recent weight change, changes in food intake, nutrition-impact symptoms that interfered with eating, and activity and functional status, while the clinician-completed component evaluated disease-related nutritional requirements, metabolic demand, and physical findings relevant to nutritional status. By integrating patient-reported and clinician-assessed information, the PG-SGA provided a multidimensional assessment of nutritional status beyond body weight alone. A total PG-SGA score of ≥4 was used to define malnutrition in this study, consistent with established clinical practice and prior literature [18,19]. Low skeletal muscle mass was defined according to the criteria of the Asian Working Group for Sarcopenia, using sex-specific SMI cutoffs (<7.0 kg/m^2^ for men and <5.7 kg/m^2^ for women), which are widely accepted for Asian populations and account for ethnic differences in body composition [20]. BMI was calculated as body weight divided by height squared (kg/m^2^).

### 2.3. Bioelectrical Impedance Analysis

Body composition was assessed using a multi-frequency BIA device (InBody 770; InBody Co., Ltd., Seoul, Republic of Korea). All BIA measurements were performed prospectively according to a predefined study protocol by a single trained nurse using the same device throughout the study period to minimize inter-observer and inter-device variability. BIA measurements were obtained at seven predefined time points: preoperatively; postoperative day (POD) 1, POD 3, and POD 5; and at 3 months, 6 months, and 12 months after surgery. Measurements were conducted with patients in the standing position following the manufacturer’s standard operating procedures. To ensure consistency, patients were instructed to avoid strenuous physical activity prior to measurement, and all assessments were performed under standardized conditions as part of routine perioperative and follow-up evaluations.

The BIA-derived parameters analyzed in this study included body weight, SMI, PhA, total body water (TBW), and extracellular water (ECW). SMI was calculated as appendicular skeletal muscle mass divided by height squared (kg/m^2^), according to Baumgartner’s definition [21]. PhA was derived from resistance and reactance values and was used as a surrogate marker of cellular integrity and membrane function. The ECW/TBW ratio was calculated as an indicator of fluid balance and systemic physiological stress.

Given the potential influence of acute postoperative fluid shifts on BIA measurements, particularly during the immediate postoperative period [22], BIA data obtained at early time points (POD 1, POD 3, and POD 5) were interpreted with caution. Accordingly, analyses focused primarily on longitudinal trends across the predefined follow-up period rather than absolute values at isolated early postoperative time points.

### 2.4. Statistical Analysis

Continuous variables are presented as mean ± standard deviation, and categorical variables are expressed as frequencies and percentages. The normality of continuous variables was assessed prior to analysis. Between-group comparisons were performed using the independent t-test for normally distributed continuous variables and the Mann–Whitney U test for non-normally distributed variables, as appropriate. Categorical variables were compared using the chi-square test or Fisher’s exact test. Given the exploratory nature of this prospective longitudinal study, analyses were primarily descriptive. Changes in body composition were illustrated graphically, with exploratory comparisons between predefined clinical subgroups at each time point. Formal repeated-measures analyses and time-by-group interaction tests were not performed; therefore, within-patient correlations were not modeled, and subgroup differences over time were not statistically tested. No adjustment was made for multiple comparisons, and all reported *p*-values are therefore nominal and should be interpreted cautiously. A two-sided nominal *p*-value of <0.05 was considered to indicate statistical significance. All statistical analyses were conducted using IBM SPSS Statistics version 25.0 (IBM Corp., Armonk, NY, USA).

## 3. Results

### 3.1. Baseline Characteristics of Patients

A total of 138 patients were included in the analysis, consisting of 77 patients with CRC and 61 patients with GC. The mean age of the cohort was 62.4 ± 9.5 years, and 92 patients (66.7%) were male. Baseline characteristics stratified by age, body weight, SMI, PhA, and ECW/TBW are presented in Table 1.

When stratified by age, patients aged ≥65 years showed a significantly lower proportion of ASA physical status class I and a higher proportion of ASA class II compared with younger patients (ASA I: 8.3% vs. 37.8%, *p* < 0.001; ASA II: 77.0% vs. 55.6%, *p* = 0.013). Older patients were also more likely to receive postoperative chemotherapy (45.8% vs. 25.6%, *p* = 0.016) and had a significantly higher prevalence of malnutrition as assessed by the PG-SGA (90.6% vs. 58.1%, *p* = 0.001). No significant differences were observed between age groups with respect to body mass index, preoperative CRP levels, or the presence of preoperative intestinal obstruction.

Patients in the high body weight group were more frequently male (82.9% vs. 50.0%, *p* < 0.001) and had a significantly higher body mass index compared with those in the low body weight group (27.18 ± 3.04 vs. 23.23 ± 5.65 kg/m^2^, *p* < 0.001). Other clinical variables, including ASA score, preoperative CRP, receipt of postoperative chemotherapy, and malnutrition status, did not differ significantly between the two groups.

Patients with high SMI were more likely to be male (95.7% vs. 39.1%, *p* < 0.001), younger (*p* = 0.014), and had a higher BMI (*p* = 0.044) compared with those with low SMI. No significant differences were observed between SMI groups in terms of ASA score, preoperative CRP levels, receipt of postoperative chemotherapy, or malnutrition status.

The high PhA group was characterized by a higher proportion of male patients (92.6% vs. 43.1%, *p* < 0.001) and a younger age (*p* = 0.001) compared with the low PhA group. In addition, patients with high PhA had a significantly lower proportion of ASA class III (2.9% vs. 15.7%, *p* = 0.007). Other baseline variables were comparable between the two groups.

Patients in the high ECW/TBW group were more frequently female (44.4% vs. 16.4%, *p* = 0.001), older (*p* < 0.001), and had a significantly lower proportion of ASA class I compared with those in the low ECW/TBW group (16.7% vs. 39.4%, *p* = 0.011). No significant differences were observed between ECW/TBW groups with respect to preoperative CRP levels, preoperative intestinal obstruction, postoperative chemotherapy, or malnutrition status.

Overall, baseline body composition parameters showed several nominally significant differences according to age, sex, and general physical status, whereas inflammatory markers and preoperative obstructive symptoms were comparable across the stratified groups.

### 3.2. Serial Changes in Weight

Serial changes in median body weight from the preoperative period to 12 months after surgery are shown in Figure 2. In the overall cohort, median body weight remained relatively stable during the immediate postoperative period up to POD 5, followed by a marked decline at 3 months, which represented the nadir across all subgroups. Thereafter, body weight gradually increased at 6 and 12 months, with varying numerical patterns across clinical subgroups.

When stratified by clinical factors, median body weight was numerically higher among male and younger patients and those with high SMI at most time points, whereas patterns according to cancer stage, chemotherapy, and nutritional status were less consistent (Figure 2A–F). At 3 months after surgery, body weight was lower than the preoperative value across all subgroups, followed by varying degrees of numerical regain. Female and older patients, patients with low skeletal muscle mass, and malnourished patients appeared to show less pronounced weight regain by 12 months, whereas patients with advanced-stage disease or those who received chemotherapy showed a larger numerical increase in median body weight between 6 and 12 months.

### 3.3. Serial Changes in Skeletal Muscle Index

Serial changes in median SMI from the preoperative period to 12 months after surgery are shown in Figure 3. In the overall cohort, median SMI gradually declined after surgery, reaching its lowest level at 3 months after surgery, followed by partial recovery at 6 and 12 months. Median SMI remained below the preoperative level at 12 months after surgery, suggesting incomplete recovery at the cohort level.

When stratified by clinical characteristics (Figure 3A–F), male, younger, high skeletal muscle mass, early-stage, non-chemotherapy, and non-malnourished patients generally had higher median SMI throughout follow-up. All subgroups showed a decline in median SMI at 3 months after surgery, followed by varying degrees of numerical recovery. At 12 months, recovery appeared less pronounced in the elderly, low skeletal muscle mass, advanced-stage, and malnourished groups, whereas their counterparts generally showed modest increases. Overall, median SMI remained below the preoperative level in most clinical subgroups despite partial recovery.

### 3.4. Serial Changes in Phase Angle

Serial changes in median PhA from the preoperative period to 12 months after surgery are shown in Figure 4. In the overall cohort, median PhA declined sharply on POD 1, representing the lowest value across most subgroups. Thereafter, PhA gradually increased during follow-up; however, median PhA did not return to preoperative levels by 12 months after surgery.

When stratified by clinical characteristics (Figure 4A–F), male, younger, high skeletal muscle mass, early-stage, non-chemotherapy, and non-malnourished patients generally had higher median PhA throughout follow-up. All subgroups showed a pronounced decrease in median PhA on POD 1, followed by varying degrees of numerical recovery. Elderly, low skeletal muscle mass, advanced-stage, chemotherapy-treated, and malnourished patients generally showed lower median PhA values during long-term follow-up. Overall, the decrease in median PhA was most prominent in the immediate postoperative period, with lower values remaining apparent in several clinically vulnerable subgroups during long-term follow-up.

### 3.5. Serial Changes in the Extracellular Water to Total Body Water Ratio

Serial changes in the median ECW/TBW from the preoperative period to 12 months after surgery are shown in Figure 5. In the overall cohort, ECW/TBW increased markedly on POD 1, reaching a peak across most subgroups. Thereafter, ECW/TBW gradually decreased over time and approached preoperative levels by 12 months after surgery, suggesting gradual normalization toward the preoperative level.

When stratified by clinical characteristics (Figure 5A–F), female, elderly, low skeletal muscle mass, advanced-stage, chemotherapy-treated, and malnourished patients generally had higher median ECW/TBW values throughout follow-up. Most subgroups showed an early increase in median ECW/TBW, followed by varying degrees of decline. Elderly, low skeletal muscle mass, advanced-stage, chemotherapy-treated, and malnourished patients appeared to show less complete normalization during follow-up. In contrast, their counterparts generally showed an earlier decline after the early postoperative period. Overall, elevated ECW/TBW remained apparent for longer in several clinically vulnerable subgroups despite gradual improvement over time.

## 4. Discussion

This prospective longitudinal study provides comprehensive insights into postoperative body composition dynamics in patients with gastric and colorectal cancer over a 12-month follow-up period. Distinct and time-dependent changes were observed across multiple body composition parameters, underscoring the complexity of physiological recovery after curative surgery. By simultaneously tracking multiple body composition domains at predefined perioperative and long-term time points, this study suggests that postoperative changes may occur asynchronously across different physiological domains. Body weight and SMI demonstrated a delayed decline, reaching their lowest levels at approximately 3 months postoperatively, followed by partial recovery at 6 and 12 months. In contrast, PhA decreased abruptly on POD 1, while the ECW/TBW peaked at the same time point, consistent with marked cellular and fluid changes during the immediate postoperative period. Descriptive subgroup patterns suggested less complete normalization of PhA and ECW/TBW among patients with advanced-stage disease or those receiving postoperative chemotherapy, which may be consistent with greater physiological vulnerability during follow-up.

### 4.1. Weight

Patients with low skeletal muscle mass demonstrated incomplete weight recovery at 12 months after surgery, with median body weight remaining approximately 7% below preoperative levels, suggesting that postoperative weight loss may persist in this clinically vulnerable subgroup. Previous studies in patients with gastric and colorectal cancer have similarly reported heterogeneous patterns of postoperative weight change, often characterized by significant early weight loss after surgery [10,15,16]. These findings support further evaluation of sustained nutritional and physical interventions, particularly for patients with low skeletal muscle mass.

In contrast, patients with advanced-stage disease or those receiving chemotherapy showed a greater numerical increase in body weight at 12 months after surgery than their counterparts. This apparent weight regain should be interpreted with caution, as it may reflect regression to the mean, intensified nutritional support targeting high-risk patients, or increases in fat mass or fluid rather than true functional or muscular recovery [23,24]. Collectively, these observations suggest that body weight regain alone may not adequately capture postoperative recovery and highlight the need for comprehensive body composition assessment when evaluating long-term outcomes.

### 4.2. Skeletal Muscle Index

Among patients with low skeletal muscle mass before surgery, median SMI remained below the preoperative level at 12 months, suggesting persistent depletion of skeletal muscle mass during long-term follow-up. This pattern suggests that skeletal muscle mass may not have fully returned to preoperative levels during the first postoperative year, and supports further investigation of perioperative strategies aimed at preserving skeletal muscle mass. Previous studies have demonstrated that prehabilitation programs incorporating nutritional optimization and physical exercise may attenuate postoperative catabolic stress, reduce postoperative complications and length of hospital stay, and improve postoperative quality of life [25,26]. Taken together, our findings align with existing evidence and support further investigation of perioperative strategies for patients with low skeletal muscle mass.

### 4.3. Phase Angle

PhA measurements provided additional insight into postoperative changes in cellular integrity. While most subgroups had their lowest PhA on POD 1, patients with advanced-stage disease or those receiving chemotherapy showed their lowest median values at approximately 3 months postoperatively. This distinct temporal pattern may be consistent with cumulative physiological stress related to disease burden and treatment exposure, rather than the effects of surgery alone [27,28]. Notably, PhA declined immediately after surgery across all groups, consistent with marked physiological changes during the early postoperative period [29]. Standard postoperative protocols often restrict oral intake during this phase [30]; therefore, these observations support further investigation of the potential role of early nutritional support, particularly in clinically vulnerable patients. However, further studies are needed to determine whether targeted nutritional interventions can modify postoperative PhA trajectories and improve clinical outcomes.

### 4.4. Extracellular Water to Total Body Water Ratio

ECW/TBW generally peaked on POD 1, consistent with acute postoperative changes in fluid balance [22]. However, in certain vulnerable subgroups—including females, the elderly, and patients with low skeletal muscle mass—the peak ECW/TBW values were observed on POD 3, suggesting that fluid-related changes may have persisted beyond the immediate postoperative period. These findings suggest that postoperative ECW/TBW patterns may vary according to baseline clinical characteristics.

Notably, patients with advanced-stage disease or those receiving chemotherapy showed relatively elevated median ECW/TBW values approximately 3 months after surgery. This pattern may reflect subacute systemic physiological stress related to disease burden and treatment exposure rather than isolated surgical effects [31]. Elevated ECW/TBW has been associated with impaired cardiopulmonary reserve and adverse clinical conditions in previous studies [32,33]. In this context, our observations support further evaluation of postoperative fluid balance in clinically vulnerable patient groups. Nevertheless, further studies are needed to determine whether ECW/TBW-guided monitoring or interventions can modify postoperative recovery trajectories and improve clinical outcomes.

### 4.5. Limitations

Several limitations should be acknowledged. First, serial measurements were analyzed descriptively without formal repeated-measures modeling; thus, within-patient correlations and time-by-group interactions were not assessed, and subgroup patterns should be regarded as hypothesis-generating. The single-center cohort and modest sample size limit generalizability and statistical power. In addition, no adjustment for multiple comparisons was applied, and all *p*-values are nominal. Second, GC and CRC were pooled without cancer-specific analyses; despite shared perioperative nutritional vulnerability [34], their risks of postoperative malnutrition may differ [35], potentially limiting cancer-specific interpretation of the observed patterns. Third, early postoperative BIA measurements may have been affected by perioperative fluid management and hydration status, and detailed information on measurement timing, food or fluid intake, and urination was unavailable. Fourth, the PG-SGA was assessed only preoperatively, and detailed longitudinal information on postoperative dietary intake, nutritional support, physical rehabilitation, length of hospital stay, and chemotherapy regimens and timing was not available. Postoperative complications were not evaluated in relation to body composition patterns; therefore, the influence of these perioperative and treatment-related factors could not be determined. Finally, associations between body composition patterns and longitudinal functional status, physical performance, quality of life, treatment tolerance, readmission, or survival were not evaluated, limiting their clinical interpretation. Residual confounding by baseline nutritional status, disease severity, postoperative complications, and other unmeasured clinical factors cannot be excluded, particularly when interpreting the observed associations with cancer stage and chemotherapy. Nevertheless, prospective serial BIA assessments at seven predefined time points over 12 months provide detailed descriptive data spanning the immediate and long-term postoperative periods.

## 5. Conclusions

This prospective longitudinal study suggests that postoperative body composition changes may occur asynchronously across multiple physiological domains in patients with gastric and colorectal cancer. While body weight and skeletal muscle index reached their nadir at approximately 3 months after surgery, PhA and ECW/TBW showed their most prominent changes during the immediate postoperative period. Clinically vulnerable subgroups, including older patients, patients with low skeletal muscle mass, patients with advanced-stage disease, and chemotherapy-treated patients, showed patterns suggestive of less complete recovery during follow-up. These prospectively collected longitudinal data provide a basis for future confirmatory studies of postoperative body composition and recovery.

## Figures and Tables

**Figure 1:**
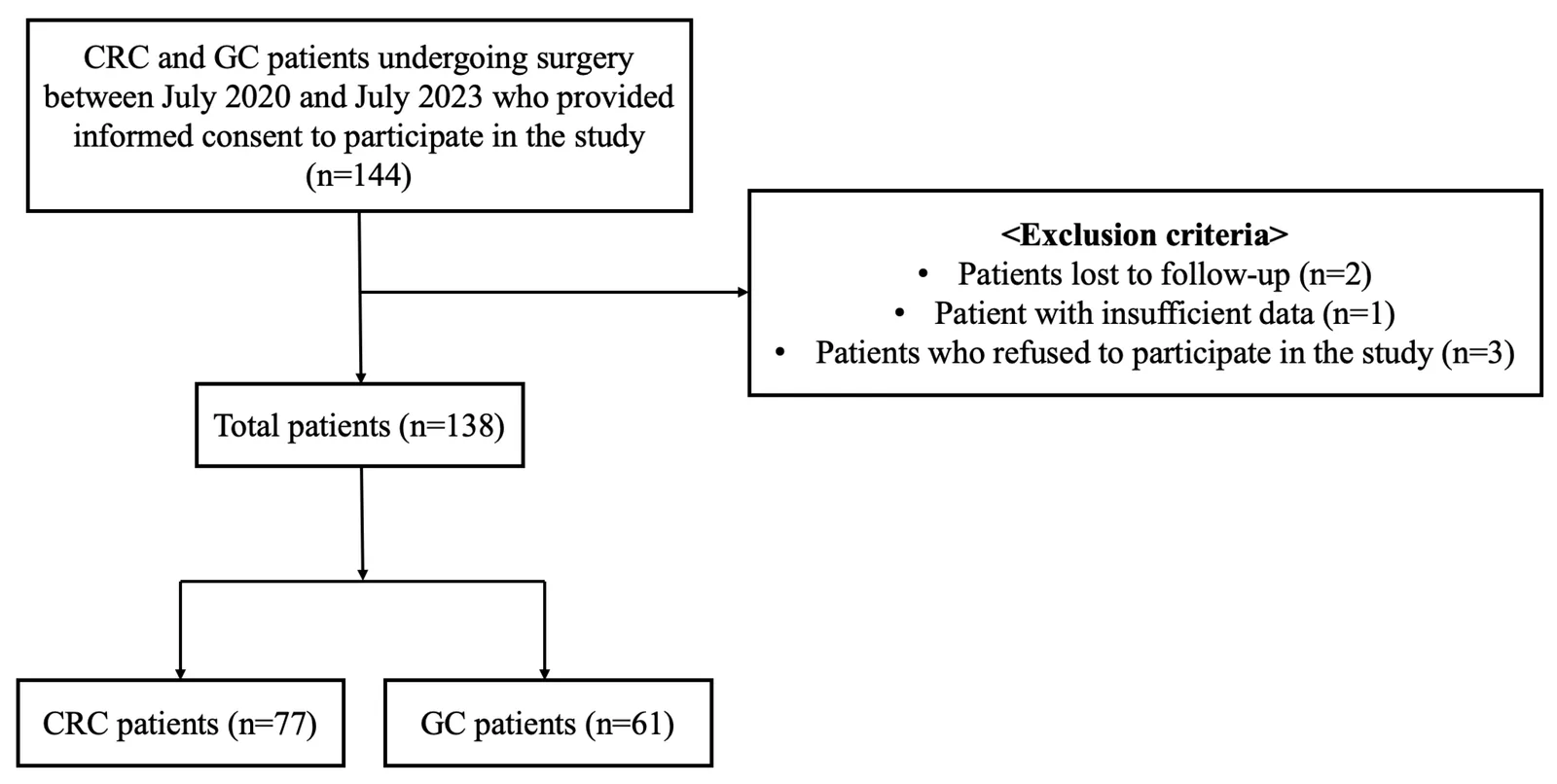
Flow diagram illustrating the selection process of patients included in the study. A total of 144 patients with CRC or GC who underwent surgery between July 2020 and July 2023 and provided informed consent were initially screened. After excluding patients lost to follow-up (*n* = 2), those with insufficient data (*n* = 1), and those who refused participation (*n* = 3), 138 patients were included in the final analysis. The study population consisted of 77 patients with CRC and 61 patients with GC.

**Figure 2:**
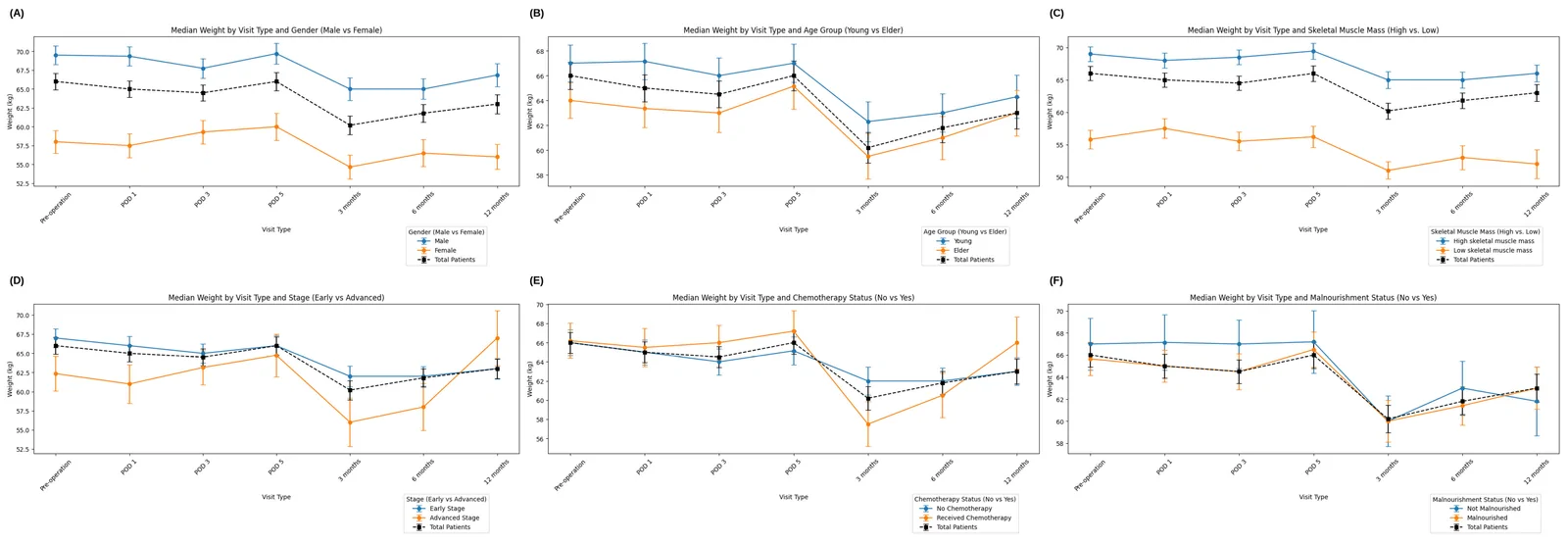
Median body weight changes from the preoperative period to 12 months postoperatively in patients with gastric and colorectal cancer, stratified by clinical factors. (**A**) Gender (Male vs. Female); (**B**) age group (Young vs. Elderly); (**C**) skeletal muscle mass (High vs. Low); (**D**) cancer stage (Early vs. Advanced); (**E**) chemotherapy status (No chemotherapy vs. Received chemotherapy); (**F**) malnutrition status (Not malnourished vs. Malnourished).

**Figure 3:**
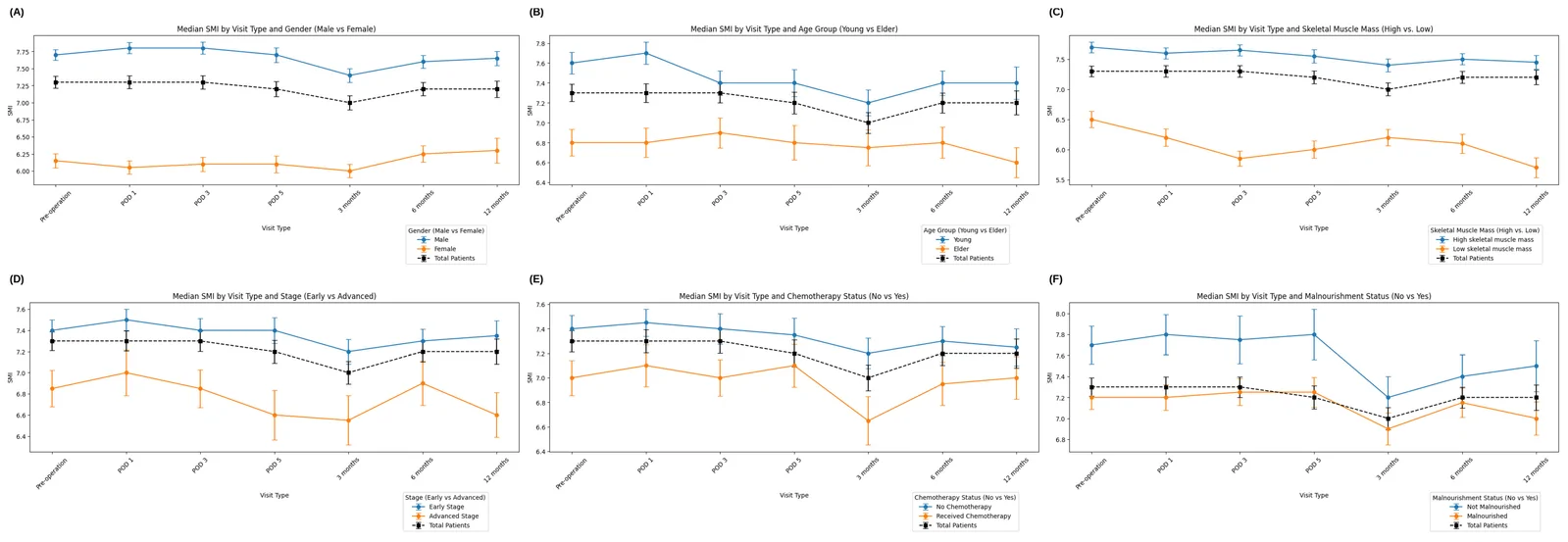
Median SMI changes from the preoperative period to 12 months postoperatively in patients with gastric and colorectal cancer, stratified by clinical factors. (**A**) Gender (Male vs. Female); (**B**) age group (Young vs. Elderly); (**C**) skeletal muscle mass (High vs. Low); (**D**) cancer stage (Early vs. Advanced); (**E**) chemotherapy status (No chemotherapy vs. Received chemotherapy); (**F**) malnutrition status (Not malnourished vs. Malnourished).

**Figure 4:**
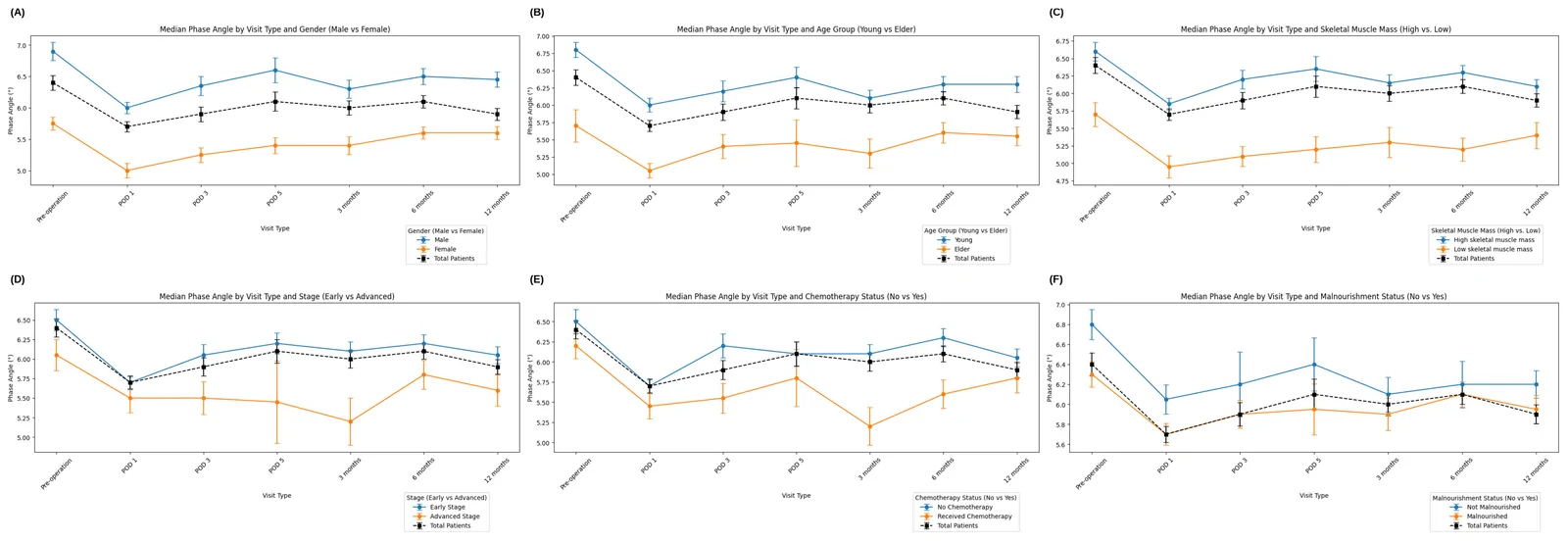
Median PhA changes from the preoperative period to 12 months postoperatively in patients with gastric and colorectal cancer, stratified by clinical factors. (**A**) Gender (Male vs. Female); (**B**) age group (Young vs. Elderly); (**C**) skeletal muscle mass (High vs. Low); (**D**) cancer stage (Early vs. Advanced); (**E**) chemotherapy status (No chemotherapy vs. Received chemotherapy); (**F**) malnutrition status (Not malnourished vs. Malnourished).

**Figure 5:**
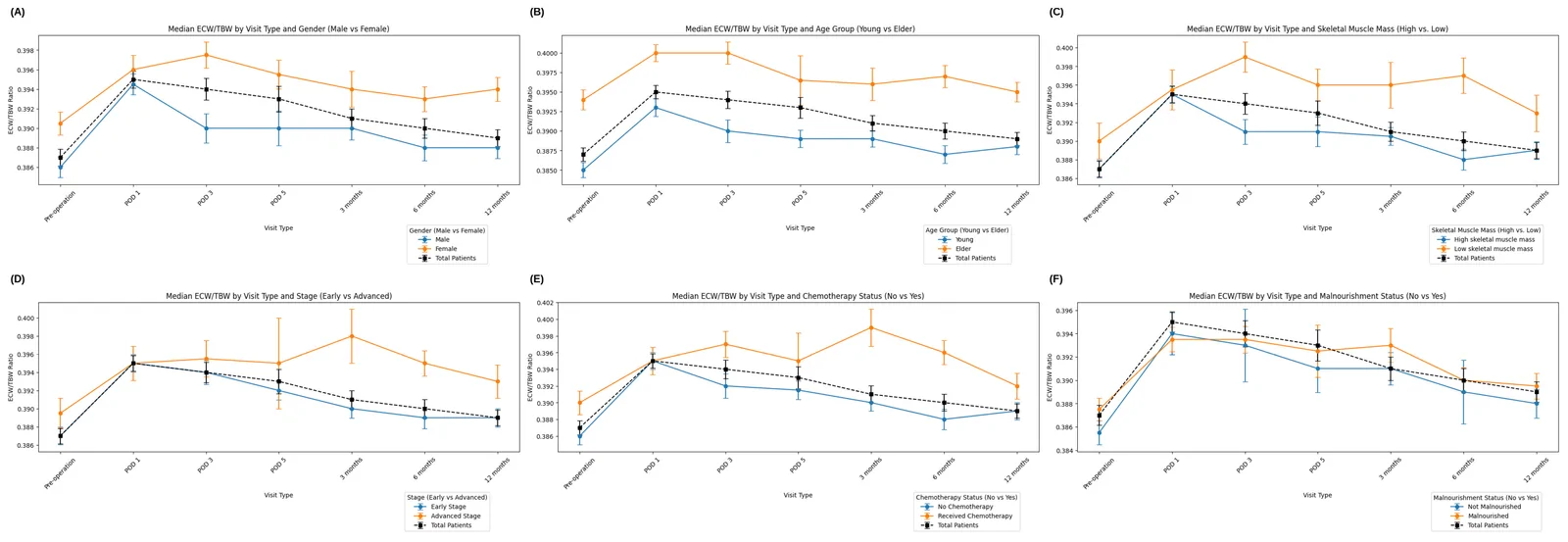
Median ECW/TBW ratio changes from the preoperative period to 12 months postoperatively in patients with gastric and colorectal cancer, stratified by clinical factors. (**A**) Gender (Male vs. Female); (**B**) age group (Young vs. Elderly); (**C**) skeletal muscle mass (High vs. Low); (**D**) cancer stage (Early vs. Advanced); (**E**) chemotherapy status (No chemotherapy vs. Received chemotherapy); (**F**) malnutrition status (Not malnourished vs. Malnourished).

**Table 1:** Patient characteristics.

	Total (*n* = 138)	Age			Weight			SMI			PhA			ECW/TBW		
		<65	≥65	*p*-Value	High	Low	*p*-Value	High	Low	*p*-Value	High	Low	*p*-Value	High	Low	*p*-Value
Sex				0.448			<0.001			<0.001			<0.001			<0.001
Male	92 (66.7)	62 (68.9)	30 (62.5)		58 (82.9)	34 (50.0)		66 (95.7)	25 (39.1)		63 (92.6)	28 (43.1)		40 (55.6)	51 (83.6)	
Female	46 (33.3)	28 (31.1)	18 (37.5)		12 (17.1)	34 (50.0)		3 (4.3)	39 (60.9)		5 (7.4)	37 (56.9)		32 (44.4)	10 (16.4)	
Age (y)	62.43 ± 9.49	57.09 ± 6.45	72.46 ± 5.12	<0.001	61.03 ± 8.46	63.88 ± 10.30	0.077	60.65 ± 7.96	64.73 ± 10.57	0.014	59.99 ± 8.16	65.37 ± 10.06	<0.001	65.94 ± 9.35	58.69 ± 8.12	<0.001
BMI (kg/m^2^)	24.69 ± 3.62	25.73 ± 6.52	24.96 ± 3.81	0.844	27.18 ± 3.04	23.23 ± 5.65	<0.001	26.02 ± 3.40	24.28 ± 6.16	0.044	25.67 ± 3.69	24.68 ± 6.03	0.250	25.60 ± 5.81	24.69 ± 3.77	0.297
Preoperative intestinal obstruction				1.000			0.620			1.000			0.358			1.000
Yes	4 (3)	3 (3.3)	1 (2.1)		3 (4.3)	1 (1.5)		2 (2.9)	2 (3.1)		1 (1.5)	3 (4.6)		2 (2.8)	2 (3.3)	
No	134 (97)	87 (96.7)	47 (97.9)		67 (95.7)	67 (98.5)		67 (97.1)	62 (96.9)		67 (98.5)	62 (95.4)		70 (97.2)	59 (96.7)	
Preoperative CRP (mg/dL)	0.25 ± 0.47	0.25 ± 0.39	0.27 ± 0.60	0.957	0.28 ± 0.55	0.23 ± 0.38	0.626	0.21 ± 0.34	0.31 ± 0.59	0.236	0.21 ± 0.33	0.31 ± 0.59	0.224	0.28 ± 0.54	0.24 ± 0.40	0.609
ASA score																
I	38 (27.54)	34 (37.8)	4 (8.3)	<0.001	17 (24.3)	21 (30.9)	0.386	17 (24.6)	21 (30.4)	0.799	21 (30.9)	17 (24.3)	0.150	12 (16.7)	26 (39.4)	0.011
II	87 (63.04)	50 (55.6)	37 (77.0)	0.013	48 (68.6)	39 (57.4)	0.172	46 (66.7)	41 (59.4)	0.615	45 (66.2)	42 (60.0)	0.709	50 (69.4)	37 (56.1)	0.210
III	13 (9.42)	6 (6.6)	7 (14.6)	0.140	5 (7.1)	8 (11.7)	0.353	6 (8.7)	7 (10.1)	0.664	2 (2.9)	11 (15.7)	0.007	10 (13.8)	3 (4.55)	0.083
Postoperative chemotherapy				0.016			0.764			0.158			0.197			0.239
Yes	45 (32.6)	23 (25.6)	22 (45.8)		22 (31.4)	23 (33.8)		19 (27.5)	25 (39.1)		19 (27.9)	25 (38.5)		27 (37.5)	17 (27.9)	
No	93 (67.4)	67 (74.4)	26 (54.2)		48 (68.6)	45 (66.2)		50 (72.5)	39 (60.9)		49 (72.1)	40 (61.5)		45 (62.5)	44 (72.1)	
Malnourished				0.001			0.326			0.092			0.205			0.053
Yes	71 (67)	43 (58.1)	29 (90.6)		35 (63.6)	37 (72.5)		34 (60.7)	38 (76)		35 (62.5)	37 (74)		42 (76.4)	30 (58.8)	
No	35 (33)	31 (41.9)	3 (9.4)		20 (36.4)	14 (27.5)		22 (39.3)	12 (24)		21 (37.5)	13 (26)		13 (23.6)	21 (41.2)	

## Data Availability

The data presented in this study are available on request from the corresponding author due to privacy.

## Abbreviations

The following abbreviations are used in this manuscript:

AJCC	American Joint Committee on Cancer
ASA	American Society of Anesthesiologists
BMI	Body mass index
BIA	Bioelectrical impedance analysis
CRC	Colorectal cancer
CRP	C-reactive protein
ECW	Extracellular water
ECW/TBW	Extracellular water-to-total body water ratio
GC	Gastric cancer
IRB	Institutional Review Board
PG-SGA	Patient-Generated Subjective Global Assessment
PhA	Phase angle
POD	Postoperative day
SMI	Skeletal muscle index
SPSS	Statistical Package for the Social Sciences
TBW	Total body water
